# Case Series of MRI and CT Assessment of Acquired Hepato-Biliary and Pancreatic Transdiaphragmatic Fistulae

**DOI:** 10.3390/tomography9040108

**Published:** 2023-07-12

**Authors:** Stefano Giusto Picchi, Giulia Lassandro, Rosita Comune, Filomena Pezzullo, Valeria Fiorini, Francesco Lassandro, Michele Tonerini, Salvatore Masala, Fabio Tamburro, Mariano Scaglione, Stefania Tamburrini

**Affiliations:** 1 Department of Radiology, Ospedale del Mare-ASL NA1 Centro, Via Enrico Russo 11, 80147 Naples, Italy; 2 Division of Radiology, Università degli Studi della Campania “Luigi Vanvitelli”, Piazza Luigi Miraglia 2, 80138 Naples, Italy; 3 Department of Radiology, Ospedale S.Anna e SS. Madonna della Neve, ASL NA3 Sud, Via Lenze, Boscotrecase, 80042 Naples, Italy; 4 Department of Emergency Radiology, Cisanello Hospital, Via Paradisa 2, 56124 Pisa, Italy; 5 Department of Medicine, Surgery and Pharmacy, University of Sassari, 07100 Sassari, Italy; 6 Department of Radiology, James Cook University Hospital & Teesside University, Marton Road, Middlesbrough TS4 3BW, UK

**Keywords:** hepato-thoracic fistula, transdiaphragmatic fistula, pancreaticopleural fistula, computed tomography (CT), magnetic resonance imaging (MRI), magnetic resonance cholangiopancreatography (MRCP)

## Abstract

Transdiaphragmatic fistulae are rare conditions characterized by pathological communication between two epithelium-lined surfaces. Hepato-thoracic fistula consists of abnormal communication between the liver and/or the biliary system and the thorax; while the pancreaticopleural fistula consists of abnormal communication between the pancreas and the thorax, the pleuro-biliary fistula represents the more common type. Clinical symptoms and laboratory findings are generally non-specific (e.g., thoracic and abdominal pain, dyspnea, cough, neutrophilia, elevated CPR, and bilirubin values) and initially, first-level investigations, such as chest RX and abdominal ultrasound, are generally inconclusive for the diagnosis. Contrast-enhanced CT represents the first two-level radiological imaging technique, usually performed to identify and evaluate the underlying pathology sustained by transdiaphragmatic fistulae, their complications, and the evaluation of the fistulous tract. When the CT remains inconclusive, other techniques such as MRI and MRCP can be performed. A prompt and accurate diagnosis is crucial because the recognition of fistulae and the precise definition of the fistulous tract have a major impact on the management acquisition process.

## 1. Introduction

Transdiaphragmatic fistulae are rare conditions characterized by pathological communications between two epithelium-lined surfaces and can be congenital or acquired [1,2,3,4]. When fistulae occur across the diaphragm, abdominal disease processes can extend into the thorax and vice versa [1]. Abdominal pathological processes that extend into the thorax can be classified anatomically as transdiaphragmatic (tumor, infection, or trauma), trans muscular (Bochdalek or Morgagni hernia), trans hiatal (e.g., stomach), intravascular (e.g., aortitis or aortic syndromes), lymphatic (hepatic hydrothorax or chylothorax), or perineural (metastasis or primary neurogenic neoplasms) [5].

### 1.1. Hepato-Thoracic Fistula

Hepato-thoracic fistula (HTF) consists of abnormal communication between the liver and/or the biliary system and the thorax, and can be classified as hepato-pleural, hepato-pulmonary, biliopleural, or broncho-biliary [6]. Biliopleural and broncho-biliary fistulae, also known as thoraco-biliary fistulae (TBF), are rare pathological entities due to abnormal communication between the biliary system and the pleural space or the bronchus, respectively [7]. TBF have been reported to be primary or congenital, or secondary to inflammation (pyogenic, amoebic, echinococcal, or tuberculosis), neoplasm, injuries (blunt or penetrant), or iatrogenic disease (after liver resection, radiofrequency ablation, bile duct stricture, irradiation, or thoracic drainage) [8]. In 1897, Graham et al. first described TBF after thoracoabdominal trauma [9]. TBF formation is considered to be more frequent than the other forms of HTF [10] because of the severe irritant effect of bile when present outside of the bile ducts or gastrointestinal tract. Secondary TBF may originate from two different pathogenic mechanisms: with or without bile duct obstruction, respectively [11,12,13]. In the case of biliary tract obstruction, bile retention can lead to a liver biloma possibly complicated by abscess formation that gradually erodes and extends through the diaphragm. All causes of biliary tract obstruction (calculi, inflammation, surgical or ablation scars, primary tumors, or metastases) can lead to this condition, especially if severe, untreated, or complicated [14]. History of a previous pleuro-parenchymal inflammatory condition can determine the extension of the fistulous tract: if the right lower lung lobe and the diaphragm are adherent for previous pleuro-parenchymal inflammatory diseases, the abscess can more easily erode the lung parenchyma reaching the nearest bronchus and determining a broncho-biliary fistula. In the absence of previous phlogistic pleuroparenchymal disease, the abscess can gradually erode and extend into the pleural space, determining a pleural empyema sustained by a biliopleural fistula [14]. This condition is present in TBF without biliary tract obstruction, such as in the case of hydatid cysts, hepatic or biliary tract neoplasms, or abscesses that gradually enlarge and erode the diaphragm, leading to a broncho-biliary fistula or biliopleural fistula [14]. Symptoms can be related to the underlying disease of the liver or the biliary tract (upper right quadrant abdominal pain irradiated to the right shoulder with jaundice in case of bile duct obstruction, fever, and weight loss), or patients can also be referred for symptoms only related to thoracic involvement such as dyspnea, cough, chest pain, and bilioptysis, which are considered to be pathognomonic for the presence of a bronchobiliary fistula [11]. Clinical presentation can be acute or chronic, but due to thoracic involvement, a fulminant scenario has been reported of patients presenting with acute respiratory distress syndrome (ARDS) [11,14]. Laboratory routine tests generally show increased white blood cell count and bilirubin in case of bile duct obstruction, and high C-reactive protein (CRP) levels [15]. The diagnosis techniques for a suspicion of TBF include the following: bronchoscopy, computed tomography (CT), and magnetic resonance imaging (MRI) with cholangiopancreatography (MRCP), and contrast-enhanced magnetic resonance cholangiography (CE-MRC), percutaneous transhepatic cholangiography (PTC), endoscopic retrograde cholangiopancreatography (ERCP), and fistulography in the case of a biliocutaneous fistula [11,14,16]. PTC, ERCP, and fistulography—in the case of biliocutaneous fistula—are considered to be the most sensitive diagnostic techniques [1]. In ER settings, chest X-rays and abdominal ultrasound are usually performed as first-line imaging with a positive detection of the right lung and hepato-biliary pathology. CT is usually performed in ER settings to determine the cause, the extension, and related complications. On CT scans, a complex fluid collection—with or without air—on the right side of the diaphragm represents a sign of potential HTF, and multiplanar reconstruction and sagittal views can usually aid in the detection of the fistulous tract. Contrast-enhanced MRI and MRCP can be useful to confirm the diagnosis given their higher accuracy compared to CT in the detection of biliary disease. CE-MRC with the use of T1w sequences after intravenous injection of hepato-biliary contrast agents (i.e., gadoxetic acid—Gd-EOB-DTPA) can be very useful in the diagnosis of a broncho-biliary fistula, because it clearly delineates the leakage of contrast agent from the biliary duct and its communication with the bronchial tree, also providing functional information about physiologic or pathologic biliary flow in addition to the display of biliary anatomy [17,18,19]. The abnormal extension of biliary signal intensity into the pleural space, lung parenchyma, or bronchi is diagnostic of TBF and allows one to define the location, size, and course of the fistula [1]. The treatment of these conditions can be surgical, conservative, or based on a combined approach [14]. Non-invasive approaches are preferred due to the lower risk of mortality and morbidity and reduced hospitalization and convalescence time compared with surgery. Surgery is the treatment of choice in chronic fistulae, consisting of complete excision of the intrathoracic and subdiaphragmatic portions of the fistula [20]. The combined approach uses biliary drainage (via ERCP or PTC) and abscess drainage (ultrasound or CT-guided) in the first step, followed by a delayed surgical intervention. This approach is required for patients who are initially not suitable for surgery (poor clinical conditions such as ARDS or sepsis) and patients where non-surgical interventions have failed [8].

### 1.2. Pancreaticopleural Fistula

Pancreatic pleural fistula (PPF) is a rare pathological entity consisting of abnormal communication between the pancreas and the thorax. PPF may originate as a rare complication of chronic pancreatitis (0.4%) [21,22]. PPF can form secondary to the leakage of pancreatic enzymes from the posterior disruption of the pancreatic duct or form an incompletely formed or ruptured pseudocyst. PPF can extend into the thorax either through the esophageal/aortic hiatus or directly through the diaphragm [1]. In addition, the pancreatic succus can dissect the fascial planes and may form direct communication either anteriorly (pancreatic peritoneal fistula) or posteriorly into the retroperitoneum; it has also been reported that retroperitoneal fluid collection can superiorly ascend and dissect into the pleural cavity, leading to PPF formation [21,23,24]. Chronic pancreatitis, related to alcohol abuse, is the more common cause of PPF in adults, while biliary duct obstruction is the more common cause in children [25,26]. Other possible causes of PPF include gallstones, abdominal trauma, and pancreatic duct anomalies [27]. Symptoms of PPF are dyspnea, cough, chest and abdominal pain, and fever [25,28]. A typical sign is pleural effusion, generally refractory to drainage and with a tendency to accumulate rapidly [21,29]. Laboratory routine tests generally show increased lipase and amylase levels, and the presence of amylase is detected in pleural fluid [23]. The diagnosis techniques in the suspicion of PPF include CT, ERCP, and MRCP, which allow the evaluation of the fistulous tract, with sensitivity of 47%, 78%, and 80% for CT, ERCP, and MRCP, respectively [28,30]. CT is considered to be less sensitive than MRI for the diagnosis and staging of pancreatitis; although in these patients with suspected thoracic involvement CT is usually the first exam performed in ER settings for high diagnostic accuracy in the diagnosis and characterization of lung involvement and for the high spatial resolution that can allow the identification of the fistulous tract, CT can also identify signs of pancreatitis (parenchymal changes, duct dilation, and fluid collection) [21,23,29]. Both ERCP and MRCP can be used to study ductal anatomy [29,31], providing information about the ampulla and ductal anatomy that may be useful in the treatment of biliary stenosis or obstruction [25,28,32]. MRCP is considered to be the imaging method of choice in PPF diagnosis [32,33], as it is a non-invasive technique, useful in the visualization of pancreatic and biliary ducts; the role of MRCP in suspected cases of PPF is to diagnose the presence and site of the fistula and to stratify further management [29,31,32].

## 2. Materials and Methods

### 2.1. Patients

Medical and surgical records of patients diagnosed with TBF and PPF at our institution were retrospectively reviewed from January 2020 to January 2022. CT, MR, and MRCP were analyzed. A total of three patients were affected by TBF and one patient was affected by PFF. Demographic data are reported in Table 1.

### 2.2. CT Imaging

CT examinations were performed using a multidetector CT system (Aquilion 64, Toshiba Medical Systems, Fukuoka, Japan) and volumetric spiral acquisition. The thickness of each slice was 1 mm, with 0.625 reconstruction, a 512 × 512 matrix, and a 40 × 40 cm FOV. Both unenhanced and contrast scans of thorax and abdomen were included in the CT protocol before and after intravenous administration of contrast agent during the arterial, venous, and delayed phases. Multiplanar reconstruction (MPR) and maximum intensity projection (MIP) were routinely used as additional diagnostic tools. About 100–130 mL of an iodinated agent, at a high iodine concentration (370–400 mg/m), was used as contrast agent, injected at 3–4 mL/s, followed by 30–40 mL of saline at the same flow rate to obtain optimal vessel depiction. We used automated bolus tracking to time the arterial phase, with the region of interest (ROI) placed in the descending aorta at an attenuation threshold of 100 HU.

### 2.3. MRI Imaging

MRI exams were conducted via a 1.5 T scanner (Amira, Siemens Medical Solutions, Erlangen, Germany) using a multichannel receive-only surface coil. Each MRI study was based on performing T1-weighted spin-echo (SE) pulse sequences in axial planes, T2 half Fourier acquisition single-shot turbo spin echo (HASTE) in axial and coronal planes, and multishot turbo spin echo sequence (BLADE) in axial planes, as well as diffusion-weighted imaging (DWI) and ADC. All patients also underwent MRCP for biliary tract evaluation.

## 3. Our Experience: Case Presentations

### 3.1. Case #1: Hepato-Thoracic Fistula

**Figure 1:**
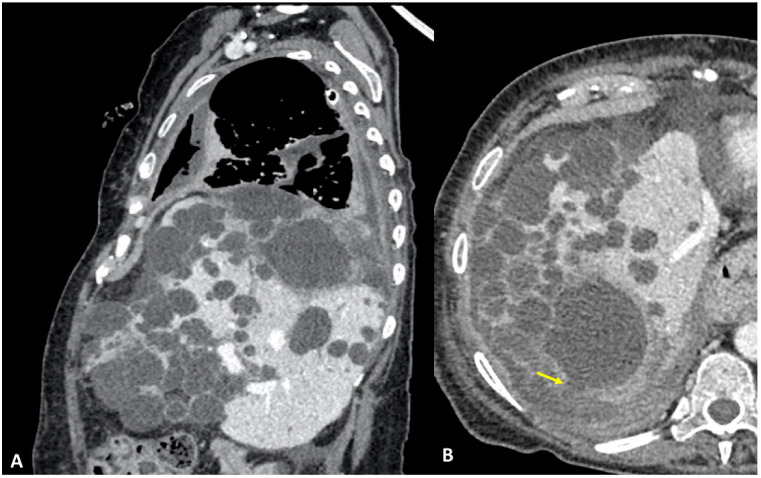
(**A**,**B**) Contrast-enhanced CT, sagittal planes (**A**), and axial planes (**B**) showing right empyema and pleural thickening with enhanced pleural leaflets. In both figures (**A**,**B**), the right lower lung lobe and a hepatic cyst look strongly adjacent (arrow), without a definite fistulous tract.

**Figure 2:**
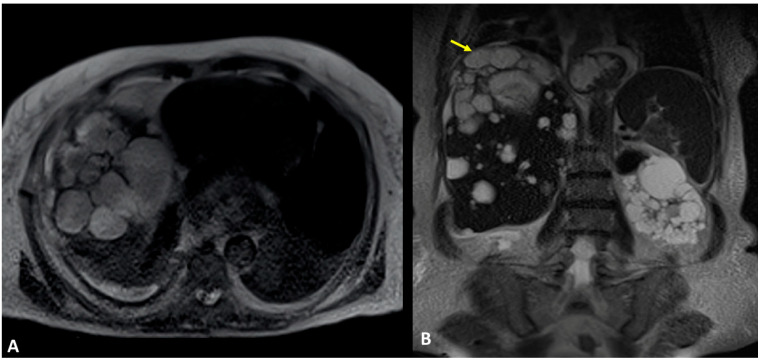
(**A**,**B**) Contrast-enhanced MRI, T2-weighted sequences, and axial (**A**) and coronal planes (**B**). In Figure (**A**), the right basal empyema and pleural thickening leaflets are confirmed. In Figure (**B**), the right lower lung lobe and a hepatic cyst (polycystosis) look strongly adjacent (arrow), without a definite fistulous tract.

A 61-year-old woman was referred to our institution in an ER setting complaining of chest pain and dyspnea. She was affected by hepatic and renal polycystosis, she previously underwent right nephrectomy, and she had been on dialysis for the last 4 years for chronic renal failure. Physical examination revealed diminished breath sounds over the right hemithorax. Laboratory routine tests showed mild neutrophilia, increased CRP levels, and a slight increase in direct bilirubin and creatinine. She underwent a chest X-ray showing a right pneumothorax (PNX) that was drained. Consequently, the patient underwent contrast-enhanced thoracoabdominal. CT showed a right pneumothorax associated with enhanced pleural thickening. At the CT, the fluid component in the right lower lung lobe was inseparable from one of the right lobe hepatic lobe cysts (Figure 1A,B). Moreover, a focal defect was appreciable in the hepatic cyst and in the right hemidiaphragm that appeared in connection with the pleural fluid empyema. The CT findings were suggestive of HTF. The following day, MRCP was performed, which confirmed that one of the cysts, localized at the seventh hepatic segment, presented a focal wall discontinuity. MRCP further supported the hypothesis of a direct connection between the hepatic cyst and the pleural cavity, but did not identified the fistulous tract (Figure 2A,B).

**Figure 3:**
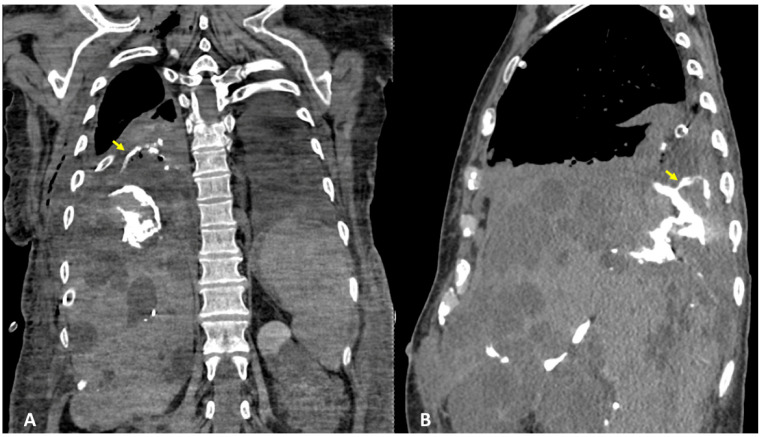
(**A**,**B**) CT performed with diluted iodinated contrast agent administered through the naso-biliar tube to obtain an exclusive enhancement of the intrahepatic biliary tract, and coronal (**A**) and sagittal planes (**B**). CT confirmed the presence of bile in a subcapsular intrahepatic cyst, showing the direct fistula with the right pleural cavity (arrows).

After the placement of the naso-biliary tube, a CT scan with diluted iodinated contrast agent administered through a tube to obtain exclusive enhancement of the intrahepatic biliary tract was performed and direct communication between the biliary branch for the 7th segment and the voluminous subcapsular intrahepatic cyst with fistulization into the right pleural cavity was detected (Figure 3A,B). The final diagnosis was TBF, and specifically biliopleural fistula sustained via the direct passage of bile into a complicated liver cyst. The patient underwent surgery for atypical lower lobe lung resection at the site of the TBF, lysis of the liver adhesions with fenestration of the cysts, and closure of the fistula.

### 3.2. Case #2: Hepato-Thoracic Fistula

**Figure 4:**
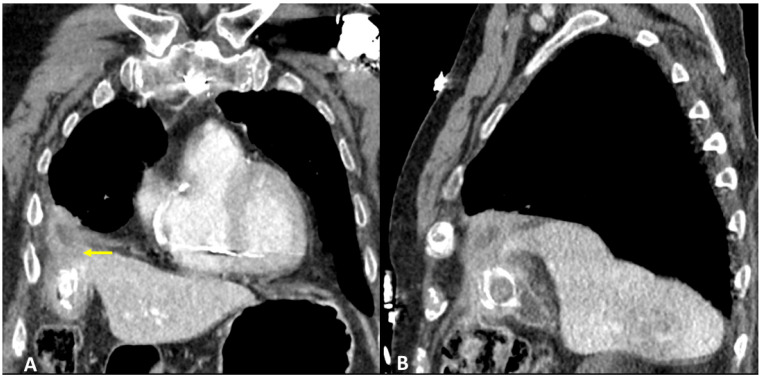
(**A**,**B**) Contrast-enhanced CT and coronal (**A**) and sagittal planes (**B**) show cholecystitis, edema, and thickening of pericholecystic adipose tissue, as well as an abscess at the gallbladder fundus adjacent to the right diaphragmatic pillar (arrow).

A 91-year-old man referred in an ER setting was complaining of acute chest pain and abdominal pain in the upper right side. The patient had diabetes, arterial hypertension, and chronic ischemic heart disease treated via aorto-coronary bypass. Physical examination revealed diminished breath sounds over the right hemithorax and a positive Murphy sign. Laboratory routine tests showed mild neutrophilia, increased CRP, and bilirubin levels. The patient underwent abdominal ultrasound showing thickened gallbladder walls, a gallstone, and the thickening of pericholecystic adipose tissue especially at the fundus where a hypoechogenic fluid collection was visualized. Chest X-ray showed right basal pleural effusion and atelectasis of adjacent lung parenchyma. The patient underwent contrast-enhanced thoraco-abdominal CT showing cholecystitis, edema, and the thickening of pericholecystic adipose tissue with small air bubbles. CT also showed an abscess at the gallbladder fundus adjacent to the right diaphragmatic pillar. Pleural effusion and parenchymal consolidation of the right lower lobe were associated with one another (Figure 4A,B). The CT diagnosed TBF, caused by cholecystitis complicated by an abscess that had eroded the right diaphragmatic pillar leading to biliopleural fistula formation. The patient was treated initially through percutaneous drainage of the pericholecystic abscess and with antibiotic therapy, followed by cholecystectomy surgery with definitive removal of the abscess remnants and closure of the transdiaphragmatic fistula.

### 3.3. Case #3: Hepato-Thoracic Fistula

**Figure 5:**
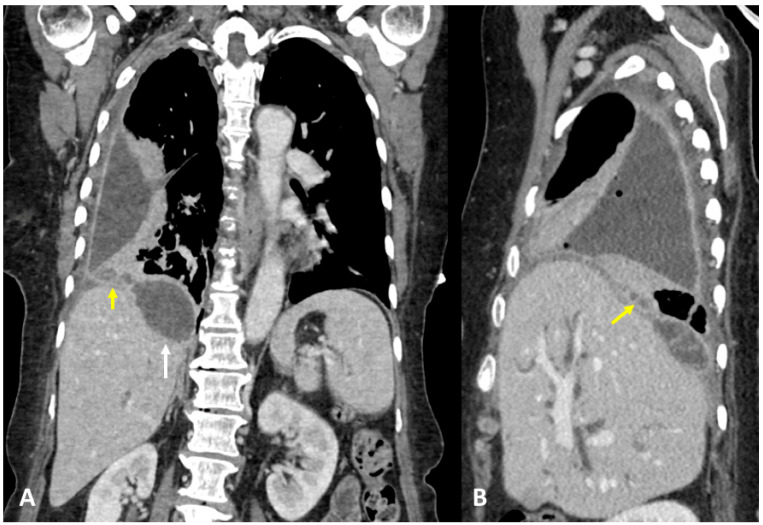
(**A**,**B**) Contrast-enhanced CT and coronal (**A**) and sagittal planes (**B**) demonstrate a right pleural empyema with gas bubbles and a collection (white arrow) located between the diaphragm and the liver. (**A**) Definite fistulous tract is noted (yellow arrows).

**Figure 6:**
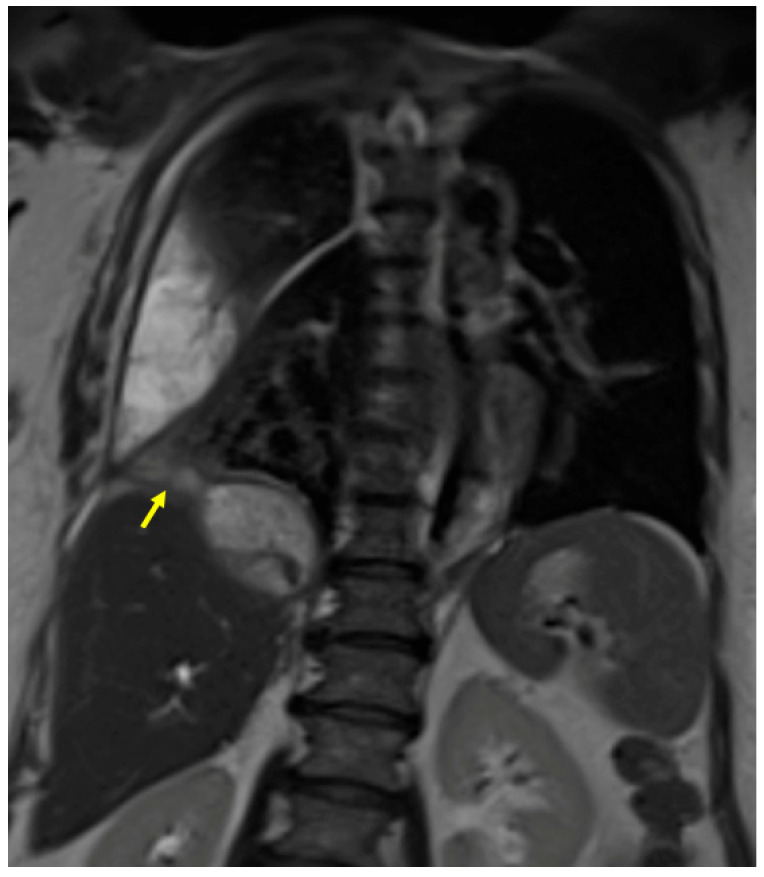
MRCP, coronal planes, and T2-weighted sequences (same patient as Figure 5). MRCP confirmed a right pleural empyema, the collection located in between the diaphragm and the liver, and the fistulous tract (arrow).

A 63-year-old woman, with no significant pathological history, was admitted to the ER, complaining about fever, cough, and abdominal pain. Physical examination revealed a positive Murphy sign. Laboratory routine tests showed mild neutrophilia and increased CRP and GT levels. The patient underwent abdominal ultrasound showing thickened gallbladder walls and gallstones, with pericholecystic fluid and biliary tract dilatation, and a hypoechogenic lesion was noted in the subdiaphragmatic liver parenchyma. Chest X-ray showed right basal pleural effusion and atelectasis of adjacent lung parenchyma. The patient underwent contrast-enhanced thoraco-abdominal CT, showing right pleural empyema with gas bubbles, cholecystitis, and gallstones, and a large collection was located in between the diaphragm and the liver. A fistulous tract was also noted (Figure 5A,B). MRCP confirmed the fistulous tract between the collection and the empyema (Figure 6). The patient underwent ERCP to remove the gallstones and to place a biliary stent. At the same time, the subdiaphragmatic collection was drained and antibiotic therapy was started. Subsequently, the patient underwent surgery for cholecystectomy, lysis of the liver adhesions, and closure of the transdiaphragmatic fistula.

### 3.4. Case #4: Pancreaticopleural Fistula

**Figure 7:**
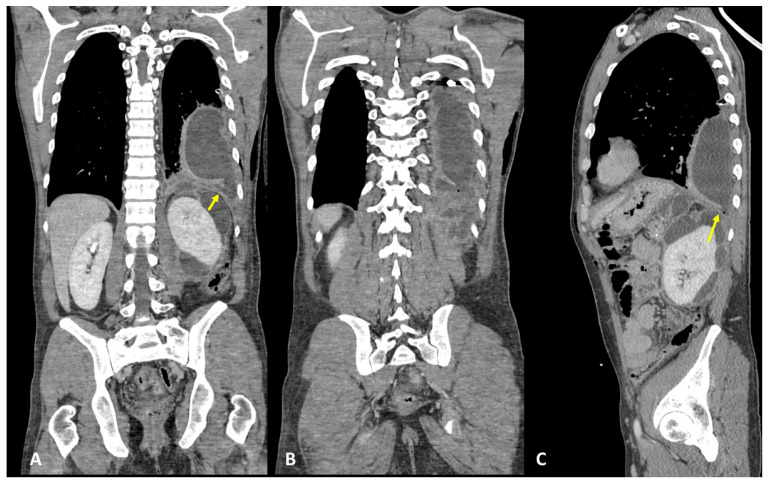
(**A**–**C**) Contrast-enhanced CT and coronal (**A**,**B**) and sagittal planes (**C**) show left pleural empyema with gas bubbles and multiple pancreatic pseudocysts located between the diaphragm, the pancreas tail, and the left kidney. A definite fistulous tract is seen (arrows).

**Figure 8:**
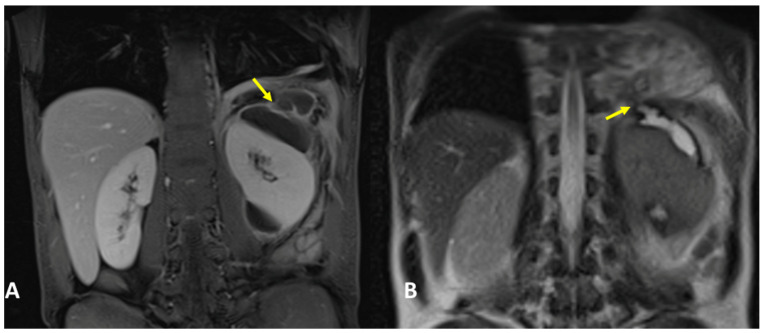
(**A**,**B**) Contrast-enhanced MRI, coronal planes, and CE T1- (**A**) and T2-weighted sequences (**B**) (same patient as Figure 7). MRI confirmed a left pleural empyema and multiple pancreatic pseudocysts located between the diaphragm, the pancreas tail, and the left kidney. The fistulous tract was detected (arrows).

A 31-year-old woman, with a history of a previous splenectomy performed 10 years ago, was referred to an ER setting with fever and diffuse thoraco-abdominal pain and was not responding to 15-day antibiotic therapy. Physical examination revealed upper intense abdominal pain and diminished breath sounds over the left hemithorax. Laboratory routine tests showed mild neutrophilia, increased CRP, and severe increase in lipase and amylase levels, notably pancreatic amylase. The patient underwent chest X-ray that showed a PNX on the left side with left lower lobe parenchymal consolidation. The pneumothorax was drained. Subsequently, the patient underwent contrast-enhanced thoraco-abdominal CT confirming left pneumothorax, left pleural empyema, and multiple communicating large pancreatic pseudocysts, with a fistulous tract through the left diaphragm (Figure 7A–C). Therefore, a diagnosis of PPF was suspected, and contrast-enhanced MRI confirmed the same findings (Figure 8A,B). The patient immediately underwent drainage of the abdominal collections and pseudocysts located in the pancreatic tail and left subdiaphragmatic lodge. Antibiotic therapy was started. In the following days, the patient underwent surgery to evacuate the empyema, pneumolysis, and placement of double pleural drainage, as well as closure of the transdiaphragmatic fistula.

## 4. Results and Discussion

Transdiaphragmatic fistula is a rare condition that can present as a thoracic extension of abdominal diseases [1,3,4]. TBF is a pathological entity classified under HTF that consists of abnormal communication between the thorax and the biliary tract, and it is associated with a high mortality and morbidity rate. Several mechanisms can result in TBF: the most frequent involves biliary tract obstruction, which can result in cholecystitis and/or cholangitis, complicated by abscess or subdiaphragmatic collection formation and pleural empyema through a fistulous tract As reported before, this may result in bile which is a strong irritant when it spreads outside of the biliary tract and gastrointestinal system [7,10]. Actually, the presence of bile in the pleural effusion or empyema is pathognomonic for a diagnosis of TBF [8]. Patients generally present with clinical vague symptoms, such as cough, fever, dyspnea, and chest and/or abdominal pain, which can be easily misinterpreted and therefore diagnosed as pneumonia or biliary colic. Diagnostic delay may result into destructive damage on pleura and lung parenchyma, with a subsequent need for decortication and wider pulmonary resection, leading to higher mortality. A high index of suspicion and a prompt diagnosis leading to early treatment are essential for minimizing the morbidity and mortality rates [7,10]. Laboratory tests typically show an elevated white blood cell count and CRP due to infectious processes that lead to fistula formation, as well as elevated levels of direct and total bilirubin in the case of bile duct obstruction in HTF [11,14]. Patients often present with non-specific symptoms and therefore initially undergo first-level investigations such as chest RX and abdominal ultrasound, which have low sensitivity for the diagnosis of TBF. These exams may also show indirect signs such as PNX, hydro-PNX, pleural effusion, parenchymal consolidation and cholecystitis, biliary tract dilatation, and cholecysto-choledochal lithiasis, respectively. Contrast-enhanced thoraco-abdominal CT is the first exam performed in ER settings in the case of suspected transdiaphragmatic fistula, due to its wide availability and high accuracy. The presence of complex fluid collections—with or without aerial nuclei—on both sides of the diaphragm represents a sign of potential TBF. CT allows the direct evaluation of the fistulous via and contrast-enhanced MRI and MRCP can be useful in confirming the diagnosis. At MRI, the abnormal extension of biliary signal intensity into the pleural space, lung parenchyma, or bronchi is diagnostic of TBF and allows to define the location, size, and course of the fistula.

PTC, ERCP, and fistulography—in the case of biliocutaneous fistula—are considered to be the most sensitive diagnostic techniques by most of the papers in the literature, while CT, MRI, and cholescintigraphy are generally considered to be less sensitive [1]. Although CT is less sensitive than the other techniques, it may lead to an earlier diagnosis of TBF, which is crucial in the management of these conditions. A delayed diagnosis can determine complications that may lead to extensive surgery and higher mortality rates [7]. The treatment of these diseases could be based on surgical, conservative, or combined approaches [14]. In TBF, treatment can be initiated conservatively via interventional endoscopy, ERCP, or drainage, and subsequently treated surgically if the conservative method is not sufficient alone [7,10,34]. The role of the combined approach is to treat the patients who are initially not candidates for surgery (cases complicated by ARDS, sepsis, or comorbidity) and patients where non-surgical interventions have failed [8,14,35]. Surgery is considered to be the treatment of choice for chronic fistulae, when non-invasive approaches fail, and for those associated with clinical deterioration, respiratory compromise, or uncontrolled sepsis. In this case, a primary transthoracic approach allowed for pleural decortications, repair of the diaphragmatic disruption, and different pulmonary resections. At surgery, the fistula tract will be entirely exposed, which necessitates the exploration of the subdiaphragmatic area and the liver, gallbladder, and biliary tract involved [7,20]. In our series of cases, the three patients affected by TBF admitted to the emergency department presented with non-specific symptoms (e.g., thoracic and abdominal pain, dyspnea, and cough) and laboratory routine test results (e.g., mild neutrophilia and elevated CPR and bilirubin values). All patients underwent first-level investigations such as chest RX and abdominal ultrasound, which showed indirect signs such as PNX, hydro-PNX, pleural effusion, parenchymal consolidation and cholecystitis, biliary tract dilatation with or without gallstones, and hypoechogenic formations suggesting abscesses. Subsequently, patients underwent contrast-enhanced CT to better delineate the chest X-ray and the abdominal ultrasound findings, due to its rapidity and ease of performance under urgent conditions. CT showed indicative or suspicious signs of TBF, such as the presence of pericholecystic and/or subdiaphragmatic collections, pleural empyema, or infected liver cysts adjacent to the diaphragm and with suspected communication with the pleural space. In one case (out of the total of three), it directly identified the transdiaphragmatic fistulous tract with the pleural space. In the other two out of three cases, the TBF was further investigated via MRI or MRCP, which better defined the site, type, and features of the TBF. Only in one case, due to the strong clinical and diagnostic suspicion of TBF, even with no definite evidence of a fistula between the infected liver cyst and the pleural cavity, the patient underwent further CT examination with a contrast agent administered into the naso-biliary tube. The contrast enhanced the intrahepatic biliary tract confirming the direct communication the suspicion of TBF fistula between the intrahepatic biliary branch and a subcapsular liver cyst which fistulated into the right pleural cavity through the diaphragm. In all three cases, antibiotic therapy was immediately started. Subsequently, two out of the three patients underwent conservative treatment by abscess drainage/placement of biliary stent/ERCP at first, and surgical treatment of cholecystectomy with the removal of residual abscesses, clearance of the pleural cavity, lysis of adhesions, and closure of the transdiaphragmatic fistula at a later stage. On the other hand, one patient underwent early surgery for atypical lower lobe lung resection, clearance of the pleural cavity, lysis of liver adhesions with fenestration of cysts, and closure of the transdiaphragmatic fistula, due to the greater severity of the clinical picture. In all three cases, survival was 100%, with longer hospitalization and convalescence time for the patient undergoing surgery directly due to the complexity of the initial clinical picture and the surgical procedure as well.

Pancreatic-pleural fistula is a rare pathological entity consisting of abnormal communication between the pancreas and the thorax. We have presented just one case of PPF; our patient was also admitted to the emergency department with vague and non-specific symptomatology (chest and abdominal pain and fever) and non-specific laboratory values (e.g., leukocytosis and increased PCR, lipase, and amylase). Therefore, after medical examination, the patient underwent chest X-ray that showed pneumothorax on the left side with left lower lobe parenchymal consolidation. The PNX was drained. Subsequently, the patient underwent contrast-enhanced thoraco-abdominal CT, confirming left pneumothorax, left pleural empyema, and multiple pancreatic pseudocysts, as well as a fistula through the left diaphragm. For further diagnostic investigation and better characterization, the patient underwent contrast-enhanced MRI, which confirmed the CT findings and allowed us to better define the site, type, and features of the PPF between the described collections and the left pleural cavity. The patient underwent antibiotic therapy and conservative treatment by draining the abdominal collections. In the following days, she underwent surgery for lysis of the empyema walls and its depletion, pneumolysis, placement of double pleural drainage, and closure of the transdiaphragmatic fistula. Survival was 100%, with relatively short hospitalization.

## 5. Conclusions

Transdiaphragmatic fistula consists of abnormal communications between the thorax and abdomen and may present as a thoracic manifestation of abdominal diseases. The identification of these diseases may require more than one imaging modality and a multidisciplinary approach for treatment. The symptoms presented by patients are generally non-specific, as are the laboratory findings, and even first-level radiological imaging exams (X-ray and ultrasound) show non-specific findings in cases of transdiaphragmatic fistula, either TBF or PPF.

Contrast-enhanced CT represents the two-level radiological imaging technique that should firstly be used, due to the chance of better evaluating the underlying pathology causing the HTF and PPF (e.g., cholecystitis and pancreatitis), the related complications (e.g., abscesses, pleural empyema, etc.), and the evaluation of any transdiaphragmatic fistula, and is strongly advised in emergency settings. However, if a CT scan does not allow a definite diagnosis, MRI, MRCP, or CEMRCP can be performed, or CT scan with contrast administration via a naso-biliary tube—if present—or percutaneous biliary drainage, in order to achieve better enhancement of the biliary tract and optimal assessment of the fistula. Imaging assessment is extremely important because it influences the management of the fistula, as conservative treatment reduces the risk of mortality, morbidity, hospitalization, and convalescence time compared with surgery, which is necessary if conservative treatment is not resolving or the patient is not improving within 72–96 hours.

## Figures and Tables

**Table 1:** Demographic data of the patients included in the study in ascending order of age and reporting the type of transdiaphragmatic fistula.

Patient No.	Transdiaphragmatic Fistula Type	Age	Sex
1	Pancreaticopleural	31	F
2	Thoracobiliary	61	F
3	Thoracobiliary	63	F
4	Thoracobiliary	91	M

## Data Availability

Data are available upon request.

## Abbreviations

Hepato-thoracic fistula (HTF); thoracobiliary fistula (TBF); pancreaticopleural fistula (PPF); computed tomography (CT); magnetic resonance imaging (MRI); magnetic resonance cholangiopancreatography (MRCP); percutaneous transhepatic cholangiography (PTC); endoscopic retrograde cholangiopancreatography (ERCP).
